# The *miR-370*/UQCRC2 axis facilitates tumorigenesis by regulating epithelial-mesenchymal transition in Gastric Cancer

**DOI:** 10.7150/jca.45553

**Published:** 2020-06-23

**Authors:** Dan-wen Wang, Fei Su, Tao Zhang, Tie-cheng Yang, Hua-qiao Wang, Li-jie Yang, Fen-fang Zhou, Mao-hui Feng

**Affiliations:** 1 Department of Gastrointestinal Surgery, Zhongnan Hospital of Wuhan University, Wuhan 430071, Hubei Province, People's Republic of China.; 2 Center for Clinical Medicine of Peritoneal Cancer of Wuhan, Wuhan 430071, Hubei Province, People's Republic of China.; 3 Department of Oncology, The First Hospital of Lanzhou University, Lanzhou 730000, Gansu Province, People's Republic of China.; 4 The Second Clinical Medical College of Lanzhou University, Lanzhou 730030, Gansu Province, People's Republic of China.; 5 Department of Biological Repositories, Zhongnan Hospital of Wuhan University, Wuhan, People's Republic of China.; 6 Clinical Cancer Study Center of Hubei Province, Wuhan 430071, Hubei Province, People's Republic of China.; 7 Key Laboratory of Tumor Biological Behavior of Hubei Province, Wuhan 430071, Hubei Province, People's Republic of China.

**Keywords:** microRNA-370, UQCRC2, gastric cancer, tumor metastasis, epithelial-mesenchymal transition, proliferation

## Abstract

Ubiquinol-cytochrome c reductase core protein 2 (UQCRC2) is an important mitochondrial complex III subunit. This study investigated the role of UQCRC2 in gastric cancer (GC) and its upstream regulatory microRNAs (miRNAs). UQCRC2 expression levels were lower in GC tissues than non-carcinoma tissues. Furthermore, UQCRC2 levels were negatively correlated with lymph node metastasis, relapse, and tumor grade. Bioinformatics analysis predicted UQCRC2 as the target gene for *miR-370,* and this was verified in luciferase reporter assays. *MiR-370* levels were inversely correlated with UQCRC2 levels in GC. UQCRC2 overexpression suppressed GC cell migration and invasion *in vitro* and *in vivo,* whereas up-regulating *miR-370* reversed these effects. Western blotting analysis showed that *miR-370* targeted UQCRC2 and positively regulated the epithelial-mesenchymal transition (EMT) signaling pathway in GC cells. Therefore, the *miR-370*/UQCRC2 axis may regulate EMT signaling pathways to affect tumor proliferation and metastasis and is, thus, a potential target for GC treatment.

## Introduction

Gastric cancer (GC) is a frequently occurring malignancy and ranks third in terms of cancer-related deaths worldwide 1. Based on the age-of-onset analysis, the incidence of GC incidence shows an increasing trend among younger individuals 2. Great efforts have been made over the last few decades to develop clinical treatments for GC. Nevertheless, specific targets are lacking because the pathogenetic mechanism remains unelucidated at the molecular level. Therefore, it is important to identify novel biomarkers for GC treatment.

Ubiquinol-cytochrome c reductase core protein 2 (UQCRC2), a critical mitochondrial respiratory complex III subunit, has a vital role within the mitochondrial respiratory chain 3. In addition, Warburg et al. first showed that irreversible damage to oxidative phosphorylation enhances aerobic glycolysis to facilitate tumorigenesis 4. Moreover, some credible results suggest that UQCRC2 is involved in many tumors as either an oncogene or a tumor suppressor gene. Abnormal UQCRC2 expression is associated with the invasion and metastasis of several cancers, such as colorectal cancer 5, breast cancer 6 and testicular cancer 7. However, the underlying molecular mechanism by which UQCRC2 participates in GC is not fully understood.

MicroRNAs (miRNAs) are a type of small noncoding RNA that can suppress target gene translation and expression via complementary pairing with the 3′-untranslated region (3′ -UTR) of target messenger RNAs (mRNAs) 8. Furthermore, miRNAs are extensively involved in cancer occurrence and development through the modulation of a variety of pathophysiological processes 9. For example, decreased *miR-370* expression has been shown to regulate PIM1 in hepatocellular carcinoma to impact cancer cell proliferation, invasion, and migration 10. Moreover, *miR-370* has previously been reported to play an essential role as a tumor suppressor in GC by targeting EGFR 11. The above findings indicate the critical role of *miR-370* in tumor occurrence and development through the targeting of various genes. However, the miRNAs upstream of UQCRC2 remain unexplored in the context of GC.

This study was performed to examine UQCRC2 levels and function in GC. The oncogenic miRNA upstream from UQCRC2 was ascertained using bioinformatics software by analyzing GC samples collected from The Cancer Genome Atlas. As a result, *miR‐370* was found to be a new oncologic miRNA through direct binding to UQCRC2. Our findings revealed the contribution of *miR-370* to the invasion, metastasis, and epithelial-mesenchymal transition (EMT) of GC through the specific targeting of UQCRC2. Furthermore, the *miR-370*/*UQCRC2* axis was identified as a potential new target for the treatment of GC.

## Materials and Methods

### Human tissues

Human primary GC tissues and matched non-carcinoma tissues were collected from 105 patients undergoing gastrectomy at Zhongnan Hospital in Wuhan, China, between December 2012 to December 2014. All patients were diagnosed with GC based on histopathological examination and were naive to any preoperative anticancer treatment. Thirty pairs of fresh GC tissue specimens and corresponding normal para-carcinoma tissue specimens were harvested at Zhongnan Hospital of Wuhan University in 2019. Tissue samples were immediately frozen in liquid nitrogen. The tumor stage of each patient was determined, and the pathological grade was rated in accordance with the GC TNM classification system formulated by the American Joint Committee on Cancer. Among the enrolled patients, 47 were female, and 58 were male, with ages ranging from 46 to 60 (average, 52 ± 6) years. Clinicopathological data, such as age, sex, differentiation, pathology, and lymph node metastasis (LNM), were collected from each patient. The Medical Ethics Committee of Wuhan University approved the study protocol (approval number: 2015011). All patients provided informed consent prior to participation, and the study was conducted in accordance with the Declaration of Helsinki.

### Cell lines and cell cultures

Six human GC cell lines (BGC-823, AGS, MKN28, MKN45, SGC-7901, and MGC-803) and the gastric epithelial cell line GES-1 were provided by the American Type Culture Collection (ATCC, Manassas, VA, USA). Cells were cultured in RPMI-1640 (Gibco, Thermo Fisher Scientific Inc., Waltham, MA, USA) containing 10% fetal bovine serum (FBS; Gibco, Thermo Fisher Scientific Inc.), 100 µg/mL streptomycin, and 100 U/mL penicillin, under the conditions of 37 °C and 5% CO_2_.

### Tissue microarrays and immunohistochemistry

Formalin-fixed paraffin-embedded (FFPE) tissue samples of GC tissue specimens and corresponding non-carcinoma tissues from 105 GC patients were used to construct the GC tissue microarray (TMA). The TMA was generated using the Quick Ray manual tissue microarrayer system (Unitma Co., Ltd., Seoul, South Korea) at Zhongnan Hospital (Wuhan, China). In brief, each case was represented by core tissue biopsies (2 mm in diameter) from FFPE tissue blocks and arranged in a new recipient paraffin block.

FFPE blocks of TMA were cut into 4-μm-thick sections, which were then deparaffinized and rehydrated using graded alcohol solutions. UQCRC2 was detected using a mouse anti-UQCRC2 antibody (1:1,000; Abcam). Color development was accomplished by incubating with 3,3'-diaminobenzidine (Dako, Carpinteria, CA, USA). Thereafter, the sections were counterstained with Mayer's Hematoxylin (Dako, Carpinteria, CA, USA), washed with water for ten seconds, dehydrated with ethanol, and cleared in xylene. Then, two drops of the permanent Entellan new mounting medium were added (Merck, Darmstadt, Germany).

Immunostaining of UQCRC2 was then performed, and the degree of staining was examined and rated by two independent observers. UQCRC2 staining was rated on a scale of 1+ to 5+, according to the proportion of positively stained cells and the intensity of cell staining, as follows: 1+, < 10% of cells with positively stained nuclei; 2+, 10-25% of cells with positively stained nuclei; 3+, 26-50% of cells with positively stained nuclei; 4+, 51-75% of cells with positively stained nuclei; and 5+, > 75% of cells with positively stained nuclei. Scores of 1+ and 2+ indicated low UQCRC2 expression levels, and scores ≥ 3+ indicated high UQCRC2 expression levels.

### Cell transfection

The *miR‐370* mimic, a *miR‐370* inhibitor, a UQCRC2-expressing plasmid, a UQCRC2-specific small interfering RNA (siRNA), and related negative controls (NCs), were chemically synthesized (GenePharma, Shanghai, China). Lipofectamine 2000 (Invitrogen; Thermo Fisher Scientific, Inc.) was used to transfect each RNA oligonucleotide and plasmid, in accordance with the manufacturer's protocol. After transfection for 48 h, the total RNA and protein were extracted.

### Animal experiments

BALB/c nude mice, 6-8 weeks old, were provided by the Shanghai Institute of Materia Medica (Shanghai, China). Animals were raised under specific pathogen-free conditions. Lentivirus-infected GC cells over-expressing UQCRC2 or negative control cells were subcutaneously injected into each group. After 5 weeks, tumor weights and volumes were determined for each animal. The Committee on Animal Research at Wuhan University approved the experimental animal protocols.

### Dual-luciferase reporter gene assay

The UQCRC2 3′-UTR containing the *miR-370* binding site was amplified using PCR and inserted upstream of the promoter in the psiCHECK-2 vector (Promega, Madison, WI, USA). The 3′-UTR of mutant or wild-type UQCRC2 was co-transfected into cells along with miR-NC or *miR370* mimics using Lipofectamine 2000 (Invitrogen). After 48 h, luciferase activity was measured using a Reporter Assay System Kit (E1910; Promega, Beijing, China). The activity of firefly luciferase was determined relative to that of Renilla luciferase. Each experiment was performed on three independent occasions.

### Quantitative reverse transcription PCR (qRT-PCR)

TRIzol RNA Isolation Reagent (Invitrogen) was used to extract total cellular RNA, and a PrimeScript RT Reagent Kit (TaKaRa, Kusatsu, Japan) was used to synthesize cDNA. A QuantiFast SYBR Green PCR kit (Invitrogen) was used for quantitative reverse transcription PCR (qRT-PCR). Gene expression was quantified according to the 2^-ΔΔct^ method, with *GAPDH* and *U6* as the mRNA and miRNA internal controls, respectively. Three replicates were analyzed for each sample. The following primer sequences were used:

UQCRC2-forward, AATTTCGTCGTTGGGAAGTAGC;

UQCRC2-reverse, ATGAGTCTGCGGATTCTGAAAG;

GAPDH-forward, GGAGCGAGATCCCTCCAAAAT, and;

GAPDH-reverse, GGCTGTTGTCATACTTCTCATGG.

### Western blotting

Total cellular protein was extracted using RIPA lysis buffer (KeyGEN, Jiangsu, China), and the protein concentration was determined using a BCA Protein Assay Kit (Beyotime Biotechnology, Haimen, China). Proteins were separated by SDS-PAGE and transferred onto cellulose acetate membranes (0.40 μm, Immobilon). The membranes were then blocked with 5% nonfat milk (Mengniu, Shanghai, China), followed by incubation with an anti-UQCRC2 primary antibody (1:1,000, Abcam) and then a horseradish peroxidase-conjugated secondary antibody (Abcam). Image-Pro Plus 6.0 (MEDIA CYBERNETICS) software was used to analyze band intensities.

### Cell proliferation analysis

The Cell-Light 5-Ethynyl-2'-deoxyuridine (EdU) Apollo Imaging Kit (Ribobio, Guangzhou, China), colony-forming assay, and cell counting Kit-8 (CCK-8) assay (Dojindo, Gaithersburg, MD, USA) were used to measure cell proliferation according to the manufacturer's instructions. In colony-forming assays, cells were inoculated into six-well plates at a density of 1,000 cells/well and incubated at 37 °C in humid conditions. After 2 weeks, the cells were washed with PBS, fixed with 100% methanol for 30 min at ambient temperature, and stained with 0.2% crystal violet for 15 min at ambient temperature. A light microscope was used to observe and count the colonies formed.

In CCK-8 assays, GC cells (1 × 10^4^ cells/well) were inoculated in 96-well plates and incubated overnight. Following 48 h of transfection, 10 μL of CCK-8 solution was added to the culture medium in each well, and the cells were incubated for 1 h at 37 °C under 95% humidity and 5% CO_2_. A microplate reader (Bio-Rad, Hercules, CA, USA) was used to measure the absorbance at 490 nm in every well to determine the cell count.

The BeyoClick™ EdU Cell Proliferation Kit, equipped with Alexa Fluor 488 (Beyotime, Shanghai, China), was used for EdU cell proliferation assays. In brief, GC cells transfected with NC or UQCRC2-expressing plasmids were treated with 50 μM EdU for 3 h. The cells were then stained by incubation with 1× Apollo solution and 100 μL of Hoechst 33342 (Ribobio, Guangzhou, China). A fluorescence microscope (Olympus, Tokyo, Japan) was used to visualize the EdU-positive cells, and the percentage of positively stained cells was calculated as the proliferation rate.

### Wound-healing assay

Transfected cells were inoculated into 6-well plates for 36 h at 37 °C under 5% CO_2_. After reaching confluence, a sterile 20-μL pipette tip was used to wound the cell monolayer. The cells were then washed with PBS and incubated in DMEM containing 1% FBS. A Nikon inverted microscope (ECLIPSE TE-2000U), equipped with a video camera (DS-U1, Nikon), was used to capture images 0 and 24 h after wounding.

### *In vitro* invasion assay

A transwell insert pre-coated with Matrigel (Corning, Inc., Corning, NY, USA) was used to test the invasiveness of GC cells. SGC-7901 and MGC-803 cells were inoculated in 24-well plates containing 200 μL of serum-free medium and then cultured in the upper transwell chamber at 4 × 10^3^ cells/well. Complete medium containing 10% FBS (400 μL) was then added to the lower chamber, and cell invasion was performed for 48 h. Thereafter, the cells were incubated at 37 °C under humid 5% CO_2_ conditions. Cells in the upper chamber were removed, and those on the lower chamber surface were fixed and stained with crystal violet. A microscope was used to count the number of invading cells and capture images.

### Gene set variation analysis (GSVA)

To infer specific biological processes and activated pathways related to low and high UQCRC2 expression groups, we used the GSVA_1.30.0 package in R 12 to evaluate t-scores. We performed GSVA using the c2 curated signatures downloaded from the Molecular Signatures Database (MSigDB). The gene list was obtained from the GSVAdata package. Gene terms with |logFC| ≥ 0.2 and P < 0.05 were considered statistically significant.

### Statistical analyses

Prism 5.0 (GraphPad Software, Inc., San Diego, CA, USA) was used for statistical analysis. Data are expressed as the mean ± standard deviation (SD) of three independent experiments. A one-way ANOVA or Student's t-test was used to analyze cell migration and proliferation. *P* < 0.05 was considered to indicate statistical significance. Stata15.0 software (StataCorp, College Station, TX, USA) was used for log-rank tests and Kaplan-Meier survival analysis. SPSS 13.0 software (IBM, Armonk, NY, USA) was used to analyze the results.

## Results

### UQCRC2 expression decreased within GC tissues or cells and was correlated with patient prognosis

**Figure 1:**
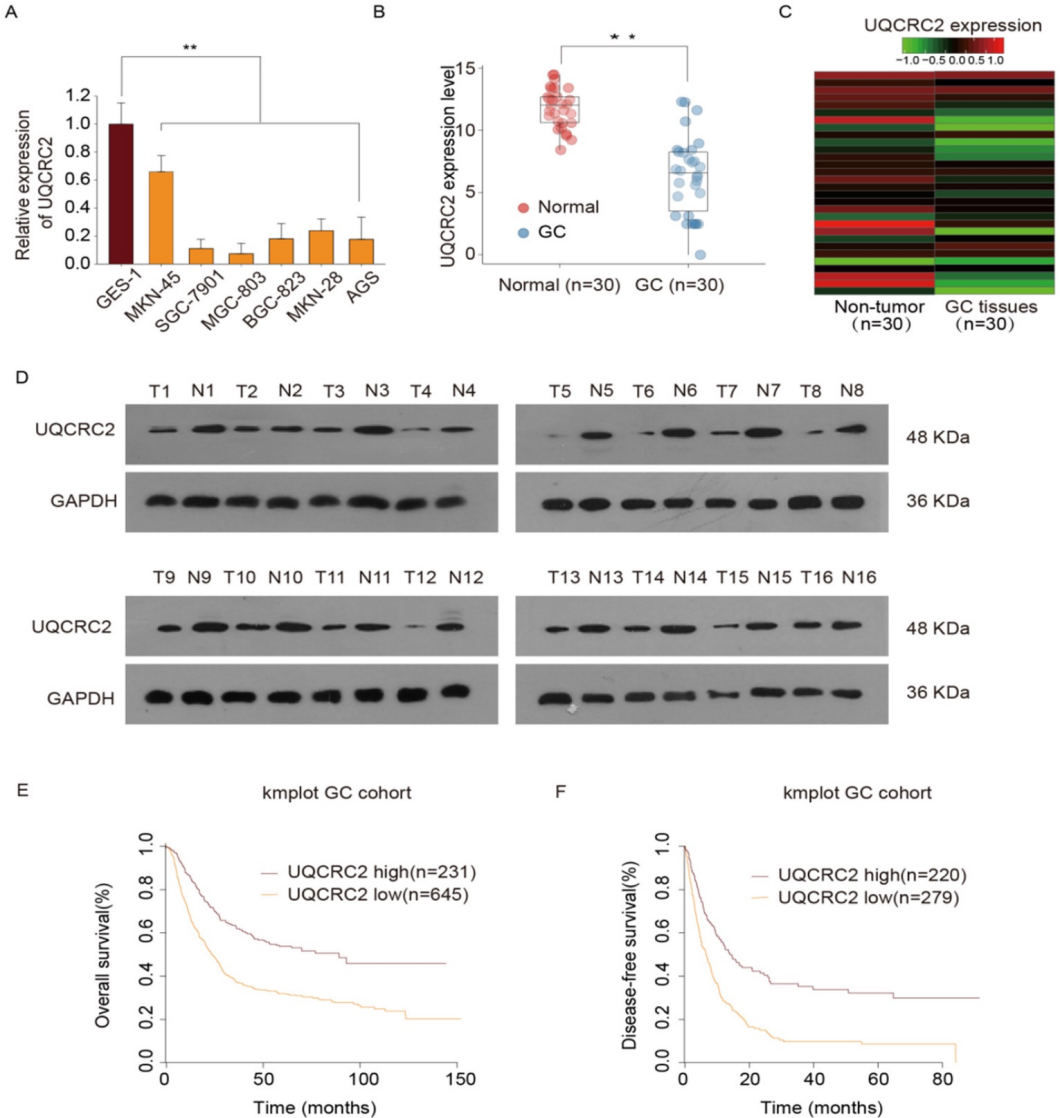
** UQCRC2 levels were down-regulated in GC tissues and cells and were correlated with superior prognosis**. (**A**) UQCRC2 expression levels in GC cells (BGC-823, AGS, MKN28, MKN45, SGC-7901, and MGC-803) and normal gastric GES-1 cells were determined by qRT-PCR. (**B**) UQCRC2 mRNA levels in 30 GC tissue specimens and matched non-carcinoma tissue specimens. (**C**) The heatmap shows UQCRC2 expression in tumor and non-tumor tissues. (**D**) Relative UQCRC2 protein expression levels among 16 pairs of randomly selected GC and normal para-carcinoma tissue specimens. (**E,F**) The Kaplan-Meier survival curve showed that patients with low UQCRC2 levels had decreased OS (HR=1.13, 95%CI [1.09‐1.18], P < 0.01) and DFS (HR=1.89, 95%CI [1.54‐2.30], P < 0.01), relative to those with high UQCRC2 expression levels. Data are expressed as the mean ± SD from three independent experiments. **P < 0.01.

In our previous study, we conducted a quantitative proteomics analysis to show that UQCRC2 expression is markedly decreased in GC. In this study, UQCRC2 expression was examined in GC cells, including BGC-823, AGS, MKN28, MKN45, and SGC-7901, MGC-803, and human gastric epithelial GES-1 cells. Relative to its expression levels in GES-1 cells and other GC cells, UQCRC2 expression levels were lower in SGC-7901 and MGC803 cells (Fig. 1A). To investigate the possible role of UQCRC2 in GC tumorigenesis, UQCRC2 levels were examined in 30 GC tissue specimens and their corresponding non-carcinoma tissue specimens using qRT-PCR. UQCRC2 expression levels were found to be decreased in primary GC (Fig. 1B-C). Additionally, UQCRC2 protein expression levels were lower in GC tissues than in the surrounding gastric tissues (Fig. 1D). To characterize the relationship between UQCRC2 and GC patient prognosis, Kaplan-Meier survival curves were plotted based on data collected from 876 GC patients in the Kaplan-Meier Plotter database (http://kmplot.com/analysis/). The downregulation of UQCRC2 was indicative of reduced overall survival (OS; hazard ratio [HR]=1.13, 95%CI [1.09‐1.18], P < 0.01) and disease-free survival (DFS; HR = 1.89, 95%CI [1.54‐2.30], P < 0.01) of patients with GC relative to the upregulation of UQCRC2 (Fig. 1E-F), suggesting the potential of using UQCRC2 to predict GC prognosis.

### UQCRC2 levels were related to the clinicopathological features of GC

**Figure 2:**
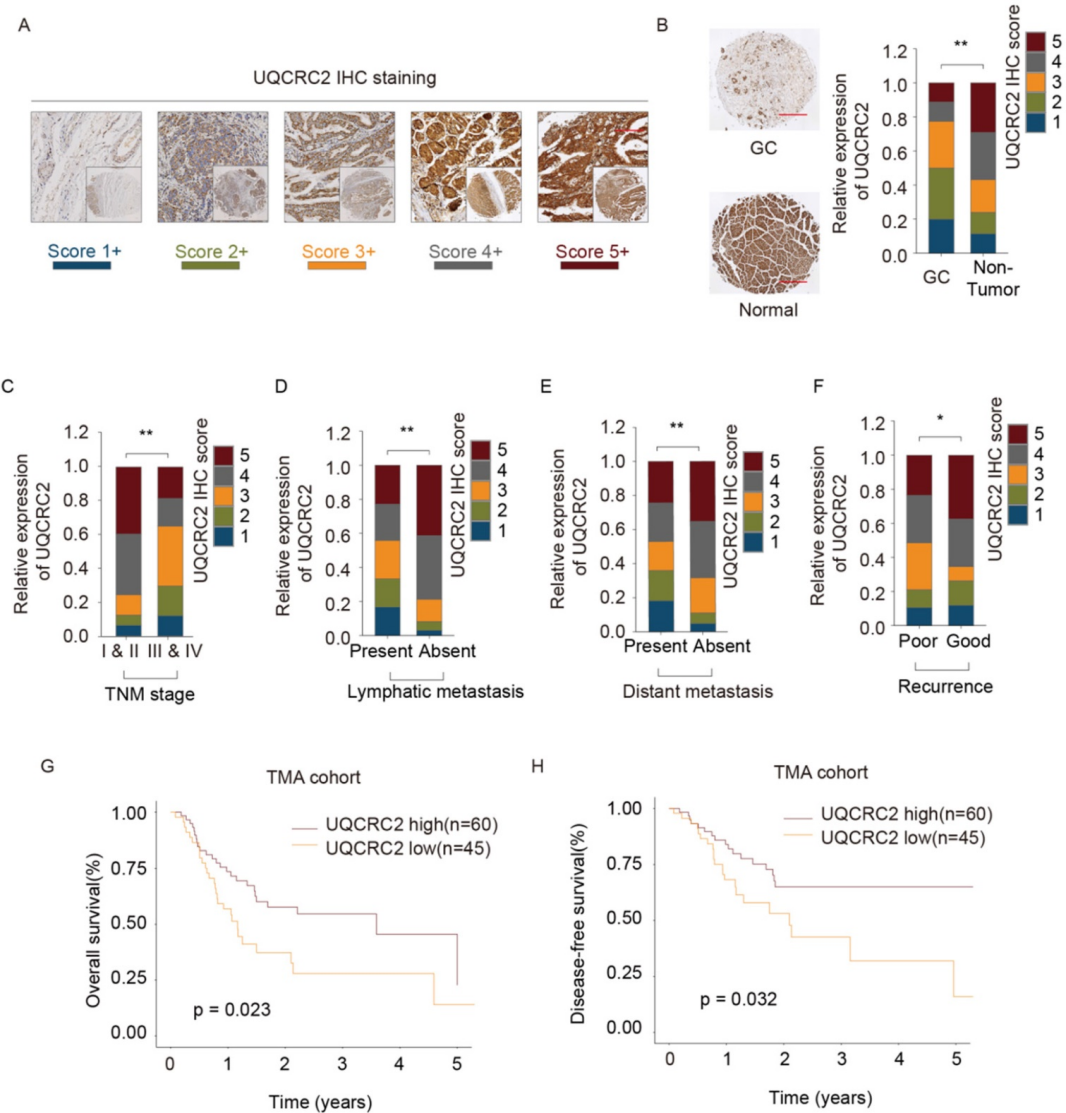
** UQCRC2 levels correlated with clinicopathological features of GC.** (**A, B**) Typical immunohistochemical staining of UQCRC2 in human GC and normal para-carcinoma tissue specimens in the tissue microarray cohort. The scores represent the intensity and frequency of UQCRC2 staining in gastric tissues. (**C-F**) Visual presentation of the relationship between UQCRC2 levels and clinicopathological features (TNM stage, lymph node metastasis [LNM], distant metastasis [DM], and relapse). (**G,H**) Over-expression of UQCRC2 was usually detected in patients with increased OS (HR=1.51, 95%CI [1.26-1.8], P < 0.05) and DFS (HR=2.09, 95%CI [1.73-2.52], P < 0.05) in the tissue microarray cohort, as suggested by Kaplan-Meier analysis.

UQCRC2 expression was analyzed in tissues from 105 GC cases to verify its relationship with clinicopathological features and its prognostic significance (Table 1). Each patient was diagnosed with GC and underwent surgical treatment. Additionally, the protein levels of UQCRC2 were found to correlate with patient clinicopathological features in the TMA cohort. Fig. 2A shows UQCRC2 IHC staining images, with scores demonstrating the intensity range and frequency of UQCRC2 staining within gastric tissues. The protein expression of UQCRC2 was markedly downregulated in tumor tissues compared to its expression in non-carcinoma tissues (Fig. 2B). UQCRC2 downregulation was significantly correlated with advanced TNM stage (*P* = 0.029), recurrence (*P* = 0.010), LNM (*P* = 0.004), and distant metastasis (*P* = 0.013, Fig. 2C-F), indicating a possible role of UQCRC2 during GC development. However, UQCRC2 expression did not correlate with other clinicopathological features in GC patients, including sex, age, and tumor diameter. Furthermore, the association of UQCRC2 protein levels with 5-year OS or DFS was examined in the TMA cohort. Patients with up-regulated UQCRC2 levels were found to have a superior OS (*P* < 0.05, Fig. 2G). Accordingly, superior recurrence-free survival was observed in patients with high UQCRC2 levels (*P* < 0.05, Fig. 2H). Therefore, the above findings suggest that UQCRC2 may function to suppress cancer development and growth, and thus, its expression levels may predict GC patient survival.

### UQCRC2 overexpression suppressed GC cell proliferation, invasion, and migration

**Figure 3:**
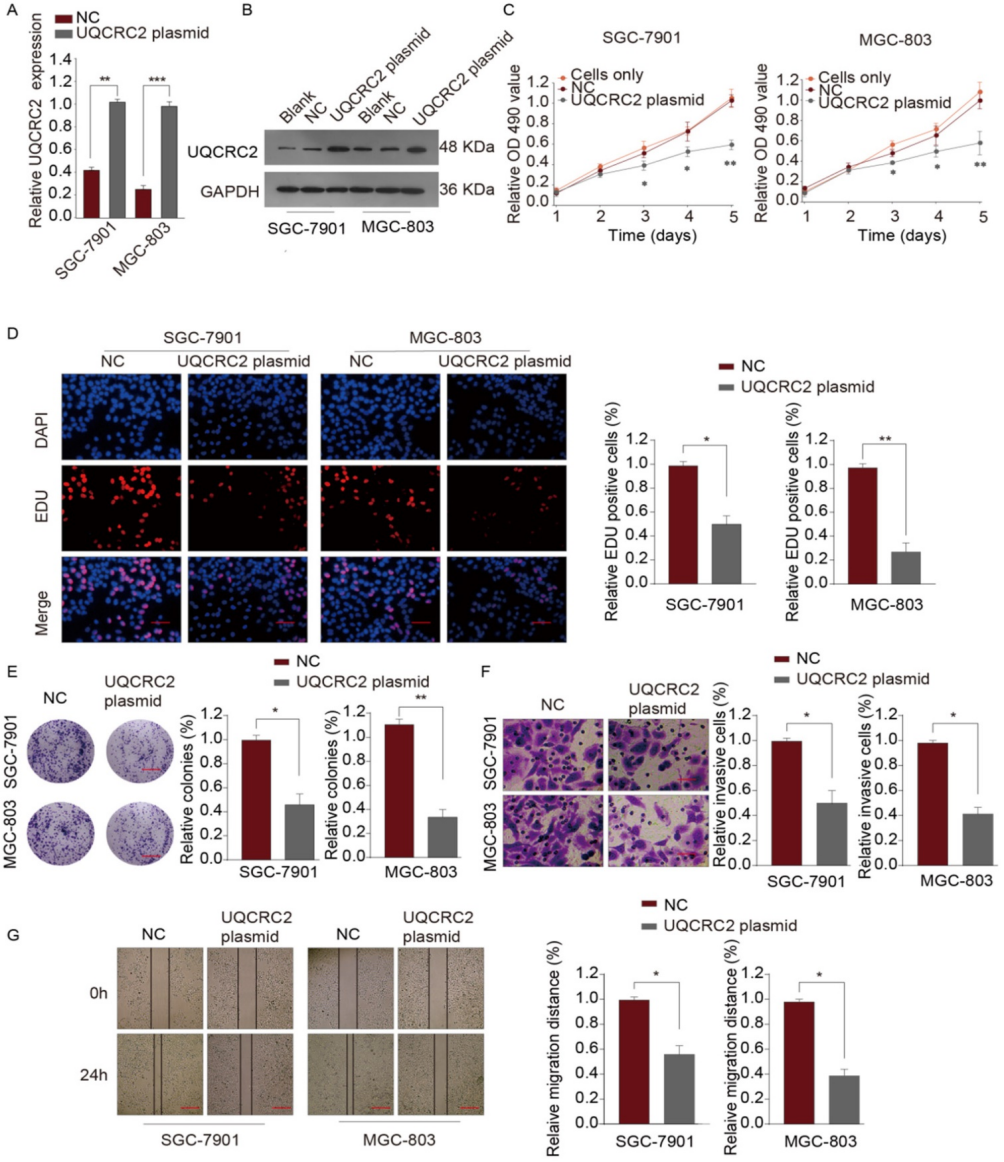
** Over-expression of UQCRC2 suppressed GC cell proliferation, invasion, and migration.** (**A, B**) The relative mRNA and protein levels of UQCRC2 in MGC-803 and SGC-7901 cells transduced with UQCRC2 plasmids and control plasmids. The blank group and cells only group are untreated cells. (**C**) Up-regulation of UQCRC2 expression suppressed MGC-803 and SGC-7901 cell proliferation, as determined using a CCK-8 assay. (**D**) MGC-803 and SGC-7901 cells were subjected to EdU staining. Typical images of EdU-stained proliferating cell nuclei (blue) and DAPI-stained cell nuclei (red) and merged images are shown. Magnification bar = 20 µm. The proportion of EdU-positive cells was plotted, which suggested that the over-expression of UQCRC2 markedly inhibited SGC-7901 and MGC-803 cell proliferation. (**E**) According to colony-forming assays, over-expression of UQCRC2 suppressed cell proliferation. (**F**) The invasive capacity was evaluated by a transwell chamber after GC cells infected with UQCRC2-overexpression plasmids and the controls. (**G**) Wound-scratch assays were performed to determine the effect of UQCRC2 on the migration of GC cells. Typical images and histograms are shown for the above-mentioned cells after migrating for 0 and 24 h.

To investigate the role of UQCRC in GC, qRT-PCR and western blotting assays were performed to measure the levels of UQCRC2 in five human GC cell lines (SGC-7901, AGS, BGC-823, MKN-45, and MGC-803). As shown in Fig. 1A, SGC-7901, and MGC-803 cells had downregulated UQCRC2 expression compared with the other GC cell lines. Therefore, SGC-7901 and MGC-803 cells were selected for subsequent functional analyses. UQCRC2-expressing plasmids were transfected into SGC-7901 and MGC-803 cells to up-regulate UQCRC2 expression. The resulting transfection efficiencies are shown in Fig. 3A-B. Based on colony-forming, EdU, and CCK-8 assays, over-expression of UQCRC2 significantly inhibited SGC-7901 and MGC-803 cell proliferation (*P* < 0.01, Fig. 3C-E). The effect of UQCRC2 overexpression on SGC-7901 and MGC-803 cell invasion was also examined using transwell assays, which suggested that over-expression of UQCRC2 significantly decreased the invasive cell count relative to control cells (*P* < 0.01, Fig. 3F). Similarly, wound-healing assays showed that overexpression of UQCRC2 inhibited the migratory capacity of SGC-7901 and MGC-803 cells (*P* < 0.05, Fig. 3G).

### UQCRC2 suppressed GC cell proliferation *in vivo*

**Figure 4:**
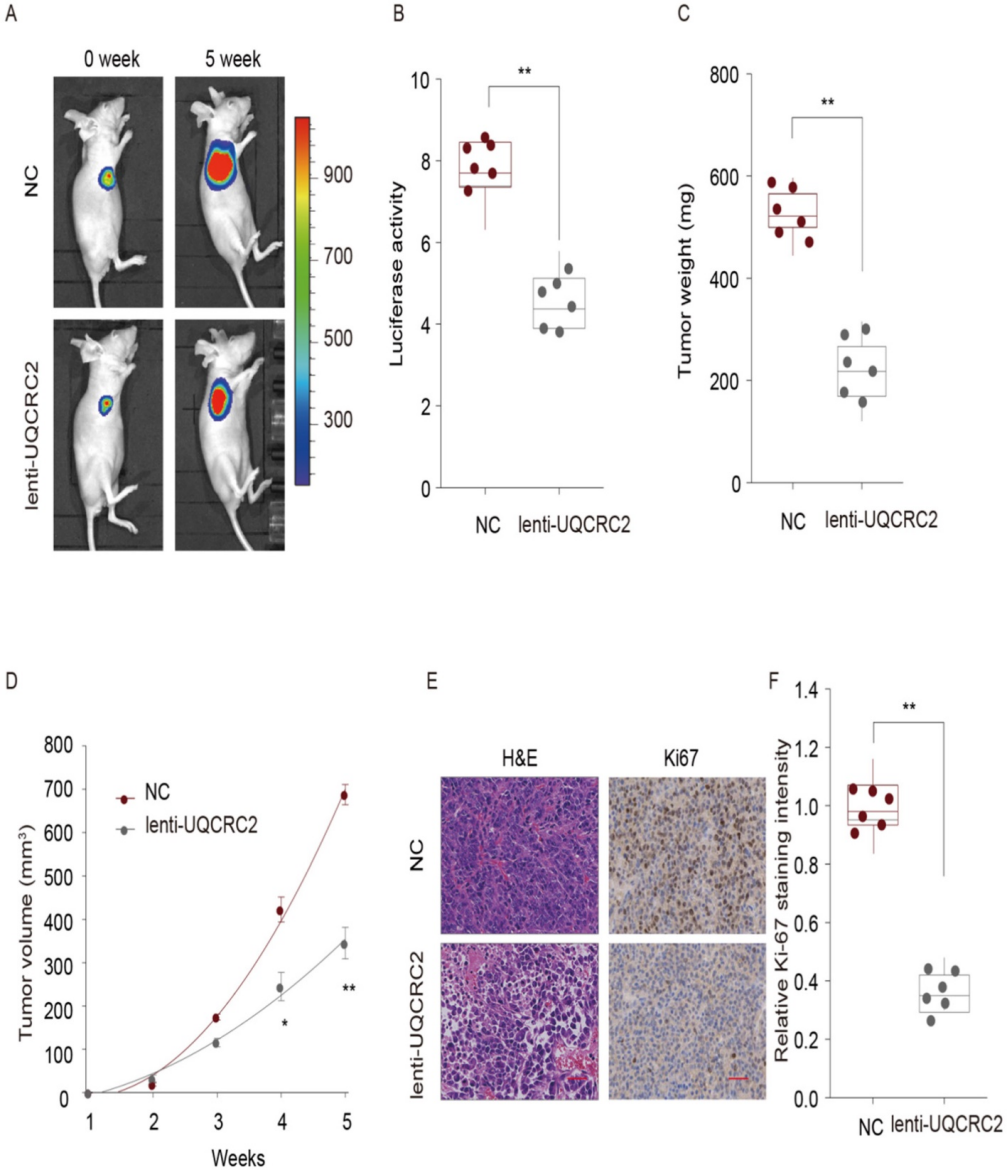
** UQCRC2 suppressed GC cell proliferation *in vivo*.** Xenograft tumor model was established in nude mice using UQCRC2-overexpressing GC cells (lenti-UQCRC2) and negative control cells. (n = 8 for each group). (**A-D**) Based on luciferase live-cell imaging, over-expression of UQCRC2 suppressed GC xenograft growth in nude mice. The volume and weight of xenograft and control cell growth were monitored. Data (mean ± SD, n = 8) were analyzed by Student's t-test; *P < 0.05, **P < 0.01. (**E**) The effect of UQCRC2 on cell metastasis was determined by Ki67 and hematoxylin and eosin staining of GC samples extracted from nude mice.

To explore the role of UQCRC2 in the proliferation of GC cells *in vivo*, a lentivirus expression plasmid was transfected into MGC-803 cells to up-regulate UQCRC2 levels. Eight male nude mice in each group were then subcutaneously injected with either UQCRC2-overexpressing GC cells (lenti-UQCRC2) or control GC cells (NC) into the right side. Five weeks later, small tumor nodules were observed in UQCRC2 over-expressing GC cells, along with reduced luciferase activity, as determined by luciferase live-cell imaging (Fig. 4A-B). The mean volume and weight of lenti-UQCRC2 tumors were lower than those of control tumors (Fig. 4C-D). As shown in Fig. 4E-F, tumors with up-regulated UQCRC2 expression exhibited decreased tumor proliferation, as evidenced by Ki67 and hematoxylin and eosin staining. These findings suggest that UQCRC2 suppresses tumorigenesis *in vivo*.

### UQCRC2 was identified as a target of* miR-370*

**Figure 5:**
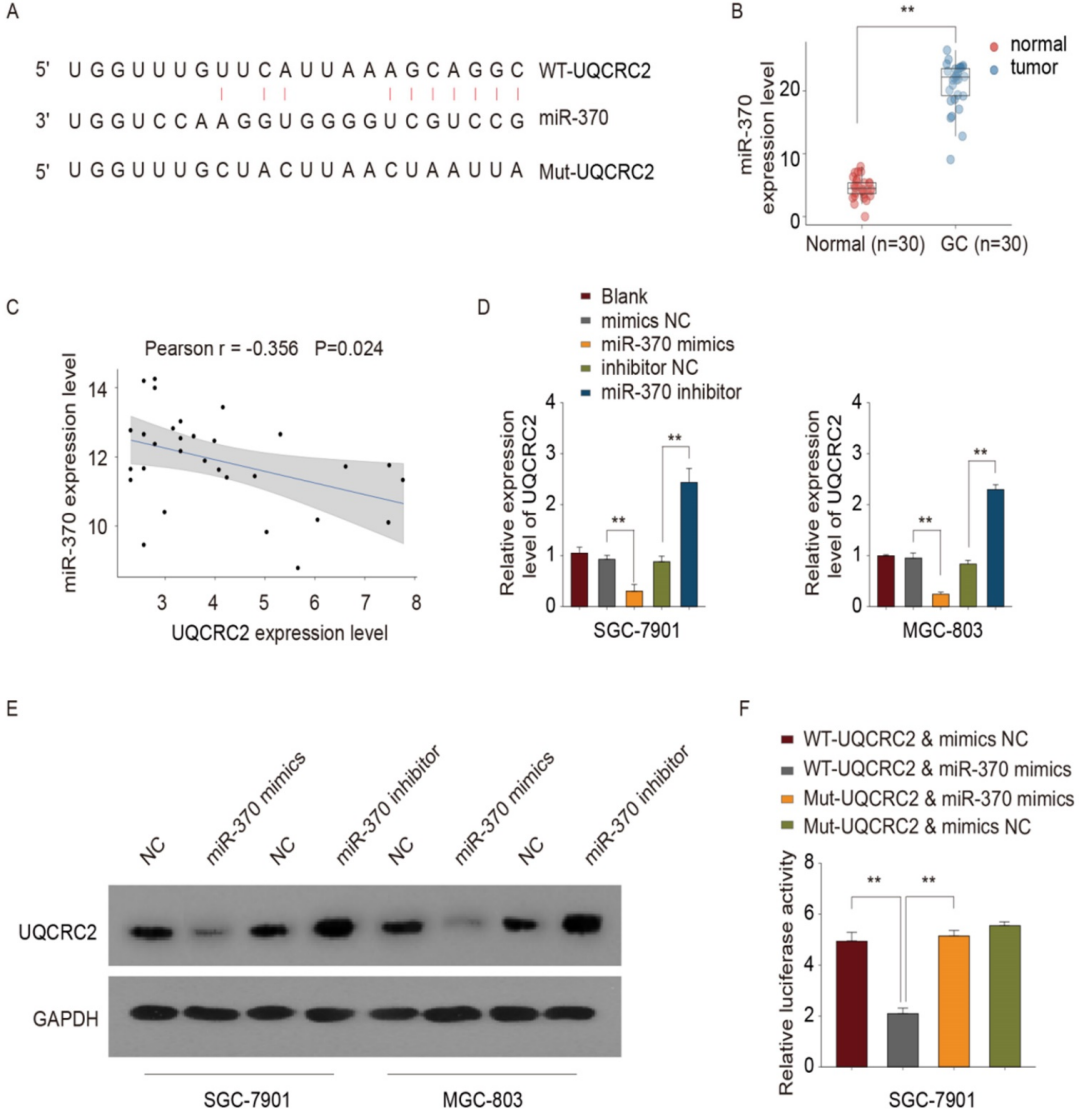
***miR-370* targeted UQCRC2.** (**A**) The microRNA.org online database was used to predict the miRNA targeting UQCRC2. (**B**) *miR-370* expression levels in 30 GC tissue specimens and matched para-carcinoma tissue specimens were measured by qRT-PCR. **P < 0.01, relative to control. (**C**) Pearson's correlation analysis showed that *miR-370* was inversely correlated with UQCRC2 mRNA levels. (**D,E**) UQCRC2 expression levels in SGC-7901 and MGC-803 cells transfected with a *miR-370* inhibitor, a *miR-370* mimic, or respective controls, were determined by qRT-PCR and western blotting. (**F**) SGC-7901 cells were co-transfected with *miR-370* mimics and plasmids expressing wild-type (WT-UQCRC2) or mutant (MUT-UQCRC2) UQCRC2. Luciferase reporter assays were performed to analyze relative luciferase activity. Data are expressed as the mean ± SD. **P < 0.01, relative to the negative control mimic.

The online database, microRNA.org, was used to predict the putative *miR-370* binding site in the 3′-UTR of UQCRC2 (Fig. 5A). Site-directed mutagenesis was used to generate UQCRC2 3′-UTR mutant constructs, to narrow down the binding site of *miR-370*. Furthermore, based on RT-PCR results, *miR-370* was up-regulated in 30 GC tissue specimens compared with corresponding non-carcinoma tissue specimens (Fig. 5B). Moreover, there was an inverse association between the *miR-370* and UQCRC2 expression levels, as indicated by Pearson's correlation analysis (r = -0.356, *P* <0.05, Fig. 5C). The introduction of *miR-370* markedly downregulated UQCRC2 expression, whereas *miR-370* knockdown up-regulated UQCRC2 expression in SGC-7901 and MGC-803 cells (Fig. 5D-E). To further confirm the direct target of *miR-370*, a dual luciferase assay was performed. Wild-type UQCRC2 was found to suppress reporter activity in SGC-7901 and MGC-803 cells. In contrast, the overexpression of mutant UQCRC2 had little effect on luciferase activity (Fig. 5F), revealing the direct regulation of UQCRC2 levels by *miR-370* in GC cell lines by binding to the 3′ -UTR sequence of UQCRC2.

### Down-regulation of UQCRC2 reversed the effect of *miR‑370* down-regulation on GC cell proliferation and invasion

**Figure 6:**
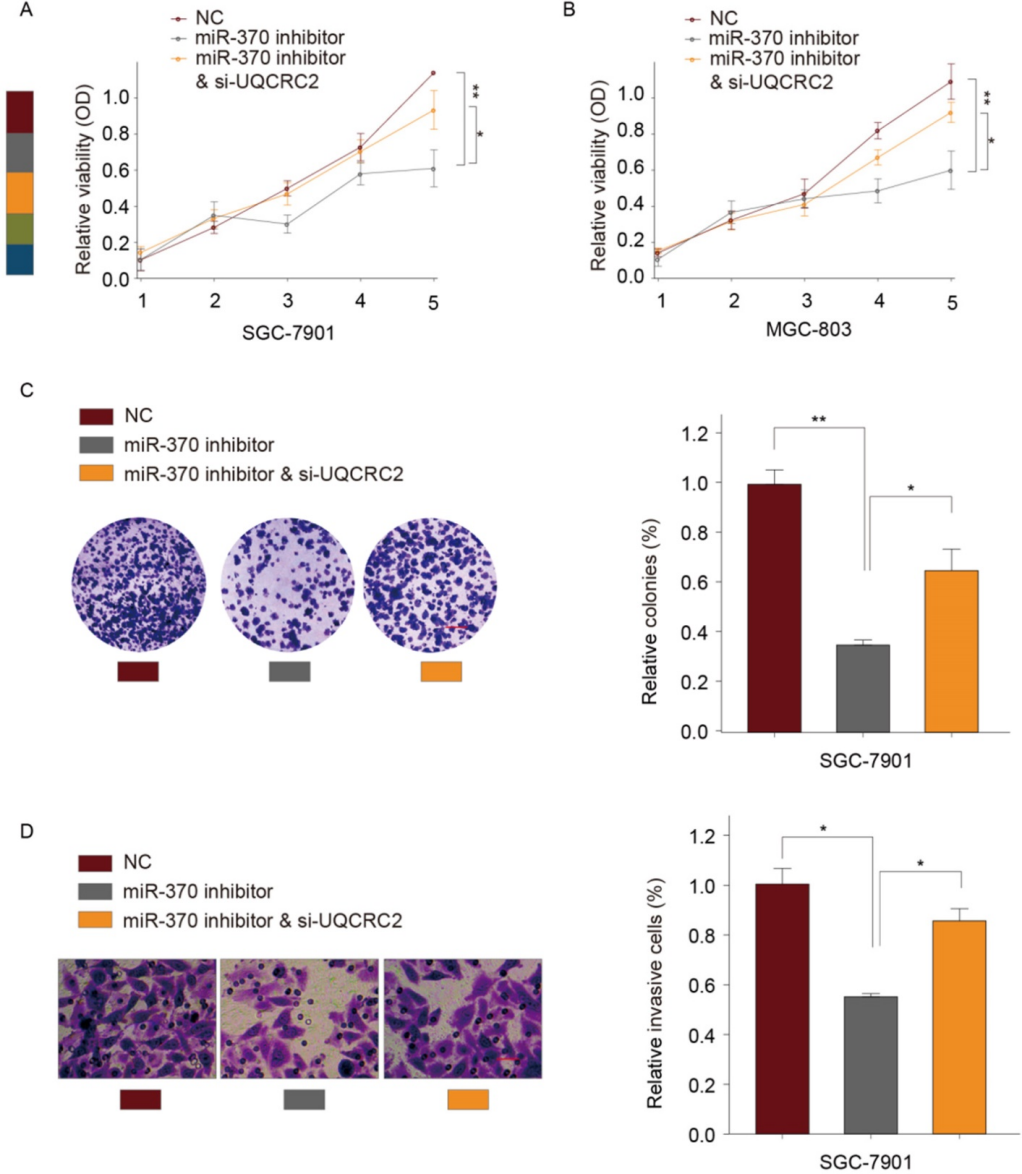
** Down-regulation of UQCRC2 expression restored the effect of *miR‑370* down-regulation on GC cell invasion and proliferation**. A UQCRC2-specific siRNA, a *miRNA-370* inhibitor, or control plasmids were transfected into MGC-803 and SGC-7901 cells. Results of CCK-8 assays of SGC-7901 (**A**) and SGC-7901 cells (**B**) and the proliferative (**C**) and invasive capacity (**D**) of SGC-7901 cells. *P < 0.05, **P < 0.01.

According to our previous experiments, overexpression of UQCRC2 markedly suppressed GC cell invasion and proliferation. To determine whether *miR-370* targets UQCRC2 to suppress GC cell migration and growth, a *UQCRC2*-specific siRNA and an *miR-370* inhibitor were co-transfected into SGC-7901 and MGC803 cells. As determined by CCK-8 assays, the simultaneous downregulation of UQCRC2 and *miR-370* reversed the *miR-370* inhibitor-induced suppression of SGC-7901 and MGC803 cell proliferation (Fig. 6A-B). Colony-forming assays yielded similar findings (Fig. 6C). According to transwell assays, the invasive capacity of SGC-7901 and MGC803 cells decreased after the downregulation of *miR-370,* and this was reversed by silencing UQCRC2 (Fig. 6D). Thus, *miR-370* may target UQCRC2 to regulate GC cell motility and viability.

### Impact of the *miR-370*/UQCRC2 axis on EMT

**Figure 7:**
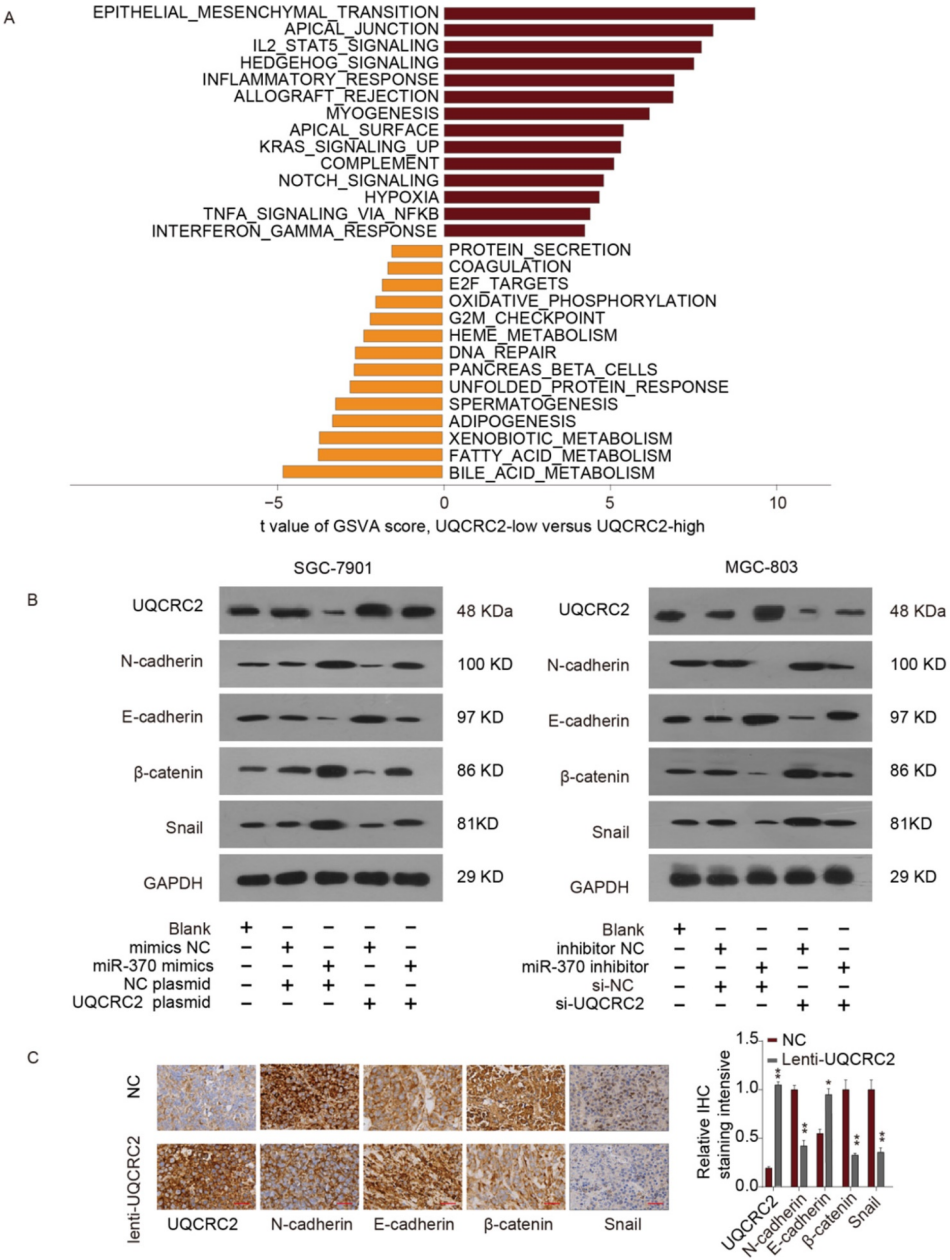
** The *miR-370*/UQCRC2 axis in epithelial-mesenchymal transition.** (**A**) The epithelial-mesenchymal transition (EMT) signal transduction pathway was estimated to be most positively correlated with EMT upon Gene Set Variation Analysis(GSVA). We set |t| > 5 as a cutoff value. (**B**) UQCRC2 and EMT marker protein expression was measured in SGC-7901 cells after transfection with a UQCRC2-expressing plasmid and *miR-370* mimics or negative control (NC) constructs. GAPDH was used as the internal reference. Western blotting analyses of the expression of UQCRC2 and EMT markers in MGC-803 cells following *miR-370* inhibitor or UQCRC2-specific siRNA transfection, relative to NC construct transfection. (**C**) Typical images of immunohistochemical staining of altered EMT hallmark proteins following UQCRC2 down-regulation.

To identify the possible *miR-370*/UQCRC2 axis mechanism, gene set variation analysis (GSVA) was performed, which revealed that the EMT signal transduction pathway was most positively correlated with EMT (Fig. 7A). According to western blotting analysis, *miR-370* mimic transfection reduced E-cadherin expression levels and restored N-cadherin, β-catenin, and Snail expression in SGC-7901 and MGC803 cells, to increase the mesenchymal phenotype. However, the effects of *miR-370* were eliminated following the transfection of a UQCRC2-expressing plasmid in the above two cell lines. On the contrary, western blotting assays also suggested that downregulation of *miR-370* markedly up-regulated E-cadherin expression and downregulated mesenchymal marker expression. These effects were reversed by co-expressing *UQCRC2*-specific siRNA (Fig. 7B). IHC staining was performed to shed more light on the effects of UQCRC2 on EMT. E-cadherin expression levels markedly decreased after down-regulating UQCRC2 in GC cells, but N-cadherin, β-catenin, and Snail were up-regulated, indicating that downregulation of UQCRC2 accelerated EMT progression (Fig. 7C). In summary, the above results indicate that *miRNA-370* directly targets UQCRC2, and the latter inhibits the EMT process in GC.

## Discussion

GC ranks as the third most common cause of cancer-related deaths worldwide, with approximately one million patients diagnosed every year 13. Recently, significant advances have been made in GC treatment, but GC patients have high relapse rates and poor prognoses 14. Therefore, the mechanisms of GC development require further investigation.

UQCRC2 is a mitochondrial respiratory complex III subunit that is up-regulated in colorectal cancer and can accelerate its proliferation and metastasis 5. Meanwhile, UQCRC2 levels are reported to be altered in many cancers, including testicular cancer and breast cancer 15-17. However, the role of UQCRC2 and specific miRNAs in the development of GC remains unclear.

Our findings revealed that UQCRC2 was downregulated in GC tissues and its expression levels correlated with certain clinicopathological features and tumorigenesis. According to large-scale functional assays, the overexpression of UQCRC2 inhibited GC pathogenesis and GC cell proliferation both *in vitro* and *in vivo*. Bioinformatics approaches were used to determine the upstream regulatory mechanism of UQCRC2, and *miR-370* was identified as a novel miRNA upstream of UQCRC2 in the context of GC.

Mature miRNAs are known to play vital roles in translational suppression and mRNA cleavage 18. *MiRNA-370* is downregulated in esophageal squamous cell carcinoma (ESCC) tissues and cells, and the downregulation of *miR-370* in ESCC affects the proliferation of cancer cells through PIN1 upregulation 19. *MiRNA-370* has previously been suggested to be associated with an increased risk of breast cancer 20. However, no studies have been performed to examine the effect of *miR-370* on UQCRC2 in GC.

This study is the first to show that *miR-370* is correlated with UQCRC2 and to demonstrate some of its downstream mechanisms. Subsequently, qRT-PCR, a luciferase reporter assay, and western blotting results verified the direct targeting of UQCRC2 by *miR-370*. Meanwhile, *miR-370* levels were increased in GC tissues and the over-expression of *miR-370* down-regulated UQCRC2 expression, whereas the downregulation of *miR-370* up-regulated UQCRC2 levels. *MiR-370* down-regulation suppressed tumorigenic signals, such as proliferation, migration, and invasion, but such effects were reversed by restoring UQCRC2 levels. These results indicate a vital role of *miR-370* in promoting GC proliferation, invasion, and migration through UQCRC2 downregulation.

EMT is widely recognized as significant in organ fibrosis, wound healing, and embryogenesis, and is particularly important in tumor metastasis 21. Increasing evidence suggests that miRNAs regulate EMT during GC pathogenesis and progression 22. Gene set variation analysis (GSVA) was performed to predict the possible regulatory pathway of the *miRNA-370*/UQCRC2 axis, and EMT was found to be the most significant downstream target of UQCRC2. Based on IHC results, UQCRC2 levels were positively correlated with E-cadherin levels and negatively correlated with N-cadherin, β-catenin, and Snail levels. The rescue assay suggested that EMT marker levels were affected by the co-transfection of GC cells with a UQCRC2-expressing plasmid and *miR-370* mimics. In summary, our results suggest that *miR-370* may target UQCRC2 to modulate EMT-related markers, but further investigation is required to determine the underlying mechanisms.

In conclusion, our results suggest that UQCRC2 and *miR-370* levels may be used to predict GC pathogenesis and metastasis. The overexpression of *miR370* enhanced GC cell proliferation, EMT, and invasion through the direct downregulation of UQCRC2 levels, indicating that the *miR-370*/UQCRC2 axis may be a novel target for the treatment of GC.

## Figures and Tables

**Table 1:** The association between UQCRC2 and clinicopathological factors in 105 GC patients in the TMA validation cohort (n=105)

Clinicopathological factors	Number (n=105)	Relative expression		P value
		Low (n=74)	High (n=31)	
**Gender**				0.608
Male	58	39	19	
Female	47	35	12	
**Age**				0.616
≤ 60	43	28	15	
> 60	62	46	16	
**Size (cm)**				0.211
≤ 5	41	36	5	
> 5	64	38	26	
**TNM stage**				0.009
I, II	59	32	27	
III, IV	46	42	4	
**Lymphatic metastasis**				0.004
NO	81	58	23	
YES	24	16	8	
**Distant metastasis**				0.013
NO	75	51	24	
YES	30	23	7	
**Recurrence**				0.051
NO	79	53	26	
YES	26	21	5	

High and low expression of UQCRC2 protein was defined according to the cut-off value of 4 for immunoreactivity score (IS).
